# The preliminary in vitro study and application of deep learning algorithm in cone beam computed tomography image implant recognition

**DOI:** 10.1038/s41598-023-45757-1

**Published:** 2023-10-27

**Authors:** Shaobo Ou-yang, Shuqin Han, Dan Sun, Hongping Wu, Jianping Chen, Ying Cai, Dongmei Yin, Huidan Ou-yang, Lan Liao

**Affiliations:** 1 The Affiliated Stomatological Hospital of Nanchang University, The Key Laboratory of Oral Biomedicine, Jiangxi Province Clinical Research Centre for Oral Diseases, Nanchang, Jiangxi Province China; 2 Information Security Evaluation Section, Jiangxi Science and Technology Infrastructure Center, Nanchang, China; 3 Vocational Teachers College, Jiangxi Agricultural University, Nanchang, China; 4 School of Stomatology, Nanchang University, The Key Laboratory of Oral Biomedicine, Jiangxi Province, Jiangxi Province Clinical Research Center for Oral Diseases, Nanchang, China; 5 Clinical Medical Research Center, Affiliated Hospital of Jinggangshan University, Medical Department of Jinggangshan University, Ji’an, Jiangxi Province People’s Republic of China; 6 The Key Laboratory of Oral Biomedicine, The Affiliated Stomatological Hospital of Nanchang University, The Affiliated Hospital of Jinggangshan University, Nanchang, Jiangxi Province China

**Keywords:** Medical research, Mathematics and computing

## Abstract

To properly repair and maintain implants, which are bone tissue implants that replace natural tooth roots, it is crucial to accurately identify their brand and specification. Deep learning has demonstrated outstanding capabilities in analysis, such as image identification and classification, by learning the inherent rules and degrees of representation of data models. The purpose of this study is to evaluate deep learning algorithms and their supporting application software for their ability to recognize and categorize three dimensional (3D) Cone Beam Computed Tomography (CBCT) images of dental implants. By using CBCT technology, the 3D imaging data of 27 implants of various sizes and brands were obtained. Following manual processing, the data were transformed into a data set that had 13,500 two-dimensional data. Nine deep learning algorithms including GoogleNet, InceptionResNetV2, InceptionV3, ResNet50, ResNet50V2, ResNet101, ResNet101V2, ResNet152 and ResNet152V2 were used to perform the data. Accuracy rates, confusion matrix, ROC curve, AUC, number of model parameters and training times were used to assess the efficacy of these algorithms. These 9 deep learning algorithms achieved training accuracy rates of 100%, 99.3%, 89.3%, 99.2%, 99.1%, 99.5%, 99.4%, 99.5%, 98.9%, test accuracy rates of 98.3%, 97.5%, 94.8%, 85.4%, 92.5%, 80.7%, 93.6%, 93.2%, 99.3%, area under the curve (AUC) values of 1.00, 1.00, 1.00, 1.00, 1.00, 1.00, 1.00, 1.00, 1.00. When used to identify implants, all nine algorithms perform satisfactorily, with ResNet152V2 achieving the highest test accuracy, classification accuracy, confusion matrix area under the curve, and receiver operating characteristic curve area under the curve area. The results showed that the ResNet152V2 has the best classification effect on identifying implants. The artificial intelligence identification system and application software based on this algorithm can efficiently and accurately identify the brands and specifications of 27 classified implants through processed 3D CBCT images in vitro, with high stability and low recognition cost.

## Introduction

For more than 40 years, dental implant system has been used as a safe and predictable treatment for replacing natural tooth roots in bone tissue, with a high survival rate of more than 90%^1–3^. The widespread use of implants makes long-term use prone to biological and mechanical complications, such as screw loosening and fracture, poor implant stability, peri-implant mucositis and peri-implanttitis^4–6^, etc. In this case, for the dentist to effectively perform necessary repairs and maintenance, they must first be able to identify specifics about the implants, such as the implant brand, the implant’s length, the diameter of the implant's post, and the type of abutment that was used.

With the continuous development of implant systems, more than 220 implant brands have been marketed since the beginning of the twentieth century^7^. Due to the increasing number of brands and types of implants worldwide, it is not easy to correctly identify implant systems^8^. In addition, when changing clinics for treatment or when the original treatment clinic closes, some patients lose their original medical records, including information about the implants. In this case, it is challenging for the doctor to determine the type of implant through an oral examination and imaging results relying solely on expertise and experience, thus making it hard for the patient to obtain precise and prompt treatment. Therefore, a method is required that can quickly and accurately identify implants with limited data.

Artificial intelligence (AI) refers to the study of machine simulation of human intelligence, thought behavior and its laws of a discipline technology. Recent advances in Internet technology have fueled AI’s rapid development and broadened its scope of possible applications. Artificial intelligence has found applications in medical imaging, diagnostic aid, etc.^9^. One of the cornerstones of artificial intelligence is deep learning, which creates neural networks to analyze and learn like the human brain. This technique allows computers to learn from data samples by training neural networks that mimic the human brain's mechanisms for recognizing patterns in information, such as text, sound and images. In deep learning research, convolutional neural networks (CNN) are most often used with excellent performance in image detection, classification, segmentation, and other analysis. It has also been successfully applied to many fields of dentistry. For example, in the identification of caries, oral dysplasia, oral cancer and other diseases have shown significant advantages^10–14^. It has been shown that using two-dimensional medical image like panoramic and apical radiographs, deep learning algorithms for implant identification are fairly accurate, and their implant identification and categorization is more accurate than that of the participating dentists^15–17^. Chaurasia A’s systematic review also showed, deep Learning models showed high accuracy in identifying and classifying dental implant systems using panoramic and periapical radiographic images^18^. However, CBCT images can avoid distortion and anamorphic of the image, while still accurately reflecting information related to the implant, making them preferable to traditional two-dimensional images. To our knowledge, there are no study using 3D CBCT images to identify implant brands and specifications by deep learning algorithms, which may be inextricably linked to the difficulty of processing and transferring 3D data. The purpose of this study is to evaluate deep learning algorithms and their supporting application software for their ability to recognize and categorize 3D CBCT images of dental implants.

## Materials and methods

### Study design

In this study, 3D CBCT images of implants in the model in-vitro were processed to obtain a two-dimensional dataset. Additionally, 9 deep learning algorithms were used to train the recognition models for the oral implant brands and specifications, such as length and diameter, with the goal of determining the best classification algorithm by comparing each algorithm's classification performance metrics.

### Material preparation

27 implant systems of various sizes from 9 brands were prepared in total (Table 1) contains details on each of them. Each implant was placed separately into the maxillary model, which used PMMA resin material and are shown Low-density images during imaging. There was no use of dental prosthetic components such as cover screws, healing abutments, or loaded prosthetics. And all CT image data were completed by the oral radiologist using CBCT (NewTom VGi), and a total of 9 sets of 3D images were taken.

**Table 1:** Implant related information.

Serial number	Brand name	Diameter (mm)	Length (mm)	Connection system
0	ABT	4.2	10.0	Conical joint (hexagon)
1	ABT	4.2	11.5	Conical joint (hexagon)
2	ABT	5.3	13	Conical joint (hexagon)
3	Astra	3.0	11.0	Conical joint
4	Astra	4.0	13.0	Conical joint
5	Astra	5.0	9.0	Conical joint
6	Nobel-active	3.5	10.0	Conical joint (hexagon)
7	Nobel-active	4.5	10.0	Conical joint (hexagon)
8	Nobel-active	5.0	12.0	Conical joint (hexagon)
9	Nobel-CC	3.5	13.0	Internal conical joint
10	Nobel-CC	4.3	13.0	Internal conical joint
11	Nobel-CC	5.0	10.0	Internal conical joint
12	SIC	3.5	7.5	Conical joint (hexagon)
13	SIC	4.5	13.0	Conical joint (hexagon)
14	SIC	5.0	11.5	Conical joint (hexagon)
15	SNUC	4.3	8.0	Conical joint (hexagon)
16	SNUC	5.0	8.0	Conical joint (hexagon)
17	Straumann	4.8	10.0	CrossFit
18	Straumann	5.0	10.0	CrossFit
19	Straumann	3.3	12.0	CrossFit
20	Dentium	4.0	10.0	Conical joint (hexagon)
21	Dentium	4.3	10.0	Conical joint (hexagon)
22	Dentium	5.0	8.0	Conical joint (hexagon)
23	Neobiotech	3.5	11.5	Conical joint (hexagon)
24	Neobiotech	4.0	11.5	Conical joint (hexagon)
25	Neobiotech	4.5	8.5	Conical joint (hexagon)
26	Neobiotech	5.0	8.5	Conical joint (hexagon)

This table shown corresponding brand names, diameters and lengths of dental implants.

(Scanning conditions: tube voltage 110 kv, tube current 3.77 mA, exposure time 3.6 s, layer thickness 0.3 mm, exposure parameters are automatic.)

### Data set preparation

**Figure 1:**
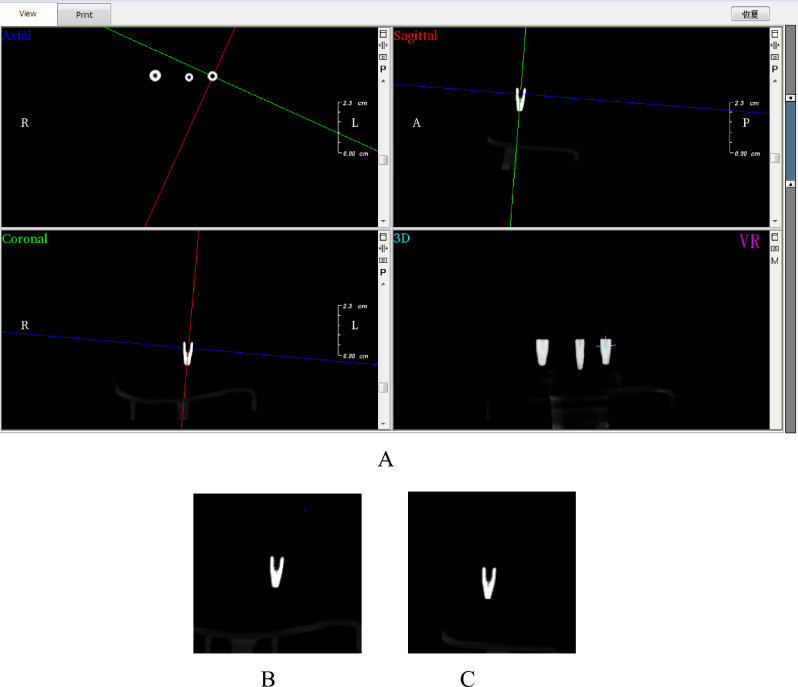
Screenshot window of CBCT. Note: (**A**) Screenshot window of CBCT with fixed Straumann 4.8–10 mm implant centric long axis unchanged. (**B**) Images in two dimensions created from coronal screenshots. (**C**) Images in two dimensions created from sagittal screenshots.

Four dentists took the two-dimensional printscreen in coronal and sagittal sections, perpendicular to the central long axis of the implant. While the central long axis of the implant remained unchanged in the CBCT image (Fig. 1A). Fixing the crop window size during printscreen will result in a 180*180 pixel resolution. Random rotations were performed on the coronal and sagittal positions, and two-dimensional printscreen with different rotation angles were taken by this method to fully obtain the implant information. 500 printscreen were taken for each implant, 250 each in coronal (Fig. 1B) and sagittal (Fig. 1C) positions, and manually identified by category according to the brand and specifications provided by the manufacturer, resulting in a final two-dimensional image dataset of 13,500 images. Once that was done, the dataset was randomly divided into a training dataset with 10,800 sheets and a test dataset with 2700 sheets.

### Deep learning algorithms

In this study, we classify the dataset using 9 deep learning algorithms, including GoogleNet, InceptionResNetV2, InceptionV3, ResNet50, ResNet50V2, ResNet101, ResNet101V2, ResNet152 and ResNet152V2. GoogLeNet is a 22-layer deep network, and its primary feature is an architecture designed to make better use of the network's available computational resources. To increase the network's depth and breadth, however, it requires a large amount of computation^19^. In order to simplify the intricate calculation, InceptionV2 introduces BN layer on InceptionV1 (GoogleNet) to avoid gradient disappearance and uses multiple small-scale convolutions instead of one large-scale convolution to reduce the number of parameters and boost computational efficiency at the same time; After the category fully connected layer, we add an LSR layer and swap out the 7 × 7 convolutional kernels for three 3 × 3 ones, Inception V3 improves upon Inception V2's computation efficiency and network classification accuracy^20^. Since increasing the depth of the network may cause gradient explosion and disappearance, based on these problems, He et al.^21^ proposed the ResNet network, a residual network, which can form a very deep network after stacking, making it possible to train very deep neural networks (NN), but the deepening of the number of layers of the network leads to its low operational efficiency. Based on the idea of whether the inception network can be as efficient when the network is deeper and wider, Sergey Ioffe et al., invented a new model, InceptionResNetV2, which is the resultant network architecture combining the Inception architecture with Residual Connections, with far better performance than the same expensive Inception network without Residual Connections. It is not only more computationally efficient than ResNet, but also has a richer network hierarchy than the Inception architecture^21,22^. Additionally, we have posted the code for the 9 deep learning algorithms' test and training datasets at https://github.com/AIdental/27-/blob/main/first%20gitHub.txt.

### Evaluation indicators

We selected accuracy rates, training time, model parameters, confusion matrix, and receiver operating characteristic (ROC) curve as the evaluation indicators for this study, in order to fully assess the classification accuracy and performance of the algorithm. With these indicators, the efficacy of this experimental algorithm in implants identification can be evaluated.

#### Accuracy rates

The degree to which the anticipated value and the actual value coincide under certain experimental circumstances is referred as the accuracy rates. The accuracy rates in this study are the proportion of properly categorized implant samples relative to the total number of implant samples and reflect the classification accuracy of the algorithm.

#### Confusion matrix

In machine learning, the confusion matrix is an error matrix that is frequently used to visually evaluate the classification accuracy of supervised learning algorithms, and the size of the confusion matrix is a square matrix of (n, n)(n: implant type). In the image accuracy evaluation, it is mostly employed to compare the categorization result to the actual value, and the accuracy of the classification result can be shown inside the matrix. The confusion matrix is determined by comparing the position and classification of each measured image elements to its corresponding value in the classified image.

#### Receiver operating characteristic (ROC) curve

Area Under Curve (AUC) is defined as the area under the ROC curve and is a performance metric that measures the quality of a classifier. As a rule, the classifier corresponding to a bigger AUC is better, hence we frequently utilize the AUC value as the model's evaluation criterion when the ROC curve is ambiguous about which classifier is superior. When AUC = 1: it represents perfect classifier, 0.5 < AUC < 1, better than random classifier, 0 < AUC < 0.5, worse than random classifier. The horizontal coordinate of the ROC curve indicates 1-specificity and the vertical coordinate indicates sensitivity, as shown in Eqs. (1) and (2) below.

1 $${\text{sensitivity}} = \frac{TP}{{TP + FN}}$$

2 $${\text{specificity}} = \frac{TN}{{TN + FP}}$$

In this study, true positive (TP) reflects the amount of implant-type samples that were accurately predicted. False negative (FN) refers to the number of samples actually predicted into other implant types for that implant type. True negative (TN), indicates the number of samples actually predicted correctly for other implant types. And False positive (FP), reflects the number of samples actually predicted into that implant for other implant types.

## Results

### Accuracy rates, training times, number of model parameters (Table 2)

**Table 2:** Summarizes the accuracy, training duration, and number of model parameters for nine algorithmic models for implant categorization.

Deep learning algorithms	Training accuracy rates	Test accuracy rates	Training times	Number of model parameters
GoogLeNet	100.0%	98.3%	1281	6,001,227
InceptionResNetV2	99.3%	97.5%	5002	54,378,235
InceptionV3	89.3%	94.8%	584	21,858,107
ResNet50	99.2%	85.4%	3087	23,643,035
ResNet50V2	99.1%	92.5%	2653	23,620,123
ResNet101	99.5%	80.7%	3877	42,713,499
ResNet101V2	99.4%	93.6%	3538	42,681,883
ResNet152	99.5%	93.2%	5370	58,426,267
ResNet152V2	98.9%	99.3%	5729	58,386,971

The batchsize and epoch trained by the 9 algorithm models tested in this study were set to 64 and 50, respectively, and in the 27 classification implant recognition, after weights were pre-trained, the training set accuracy and test set accuracy of all nine algorithms surpassed 80%.Among them, the test accuracy rate of ResNet152V2 test is 99.3%, which is the highest among the 9 algorithms and has the best classification accuracy (Table 2).

### Confusion matrix

**Figure 2:**
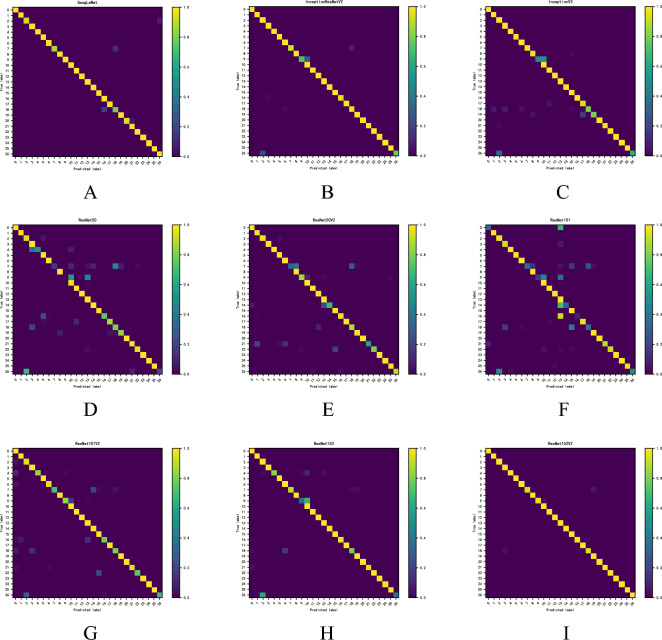
Confusion matrix results for 9 algorithms. Note: The nine algorithmic models are GoogleNet, InceptionResNetV2, InceptionV3, ResNet50, ResNet50V2, ResNet101, ResNet101V2, ResNet152 and ResNet152V2, respectively; the horizontal coordinates represent the predicted category, the vertical coordinates represent the real category, and the numbers 0–26 indicate the implant classification information as in Table 1.

Another analysis results in the implant multiclassification confusion matrix based on a dataset using 9 deep learning models (Fig. 2), and comparing the position of each measured image element with the corresponding position in the classified image yields the highest classification accuracy for the ResNet152V2 algorithm model.

### ROC curve

**Figure 3:**
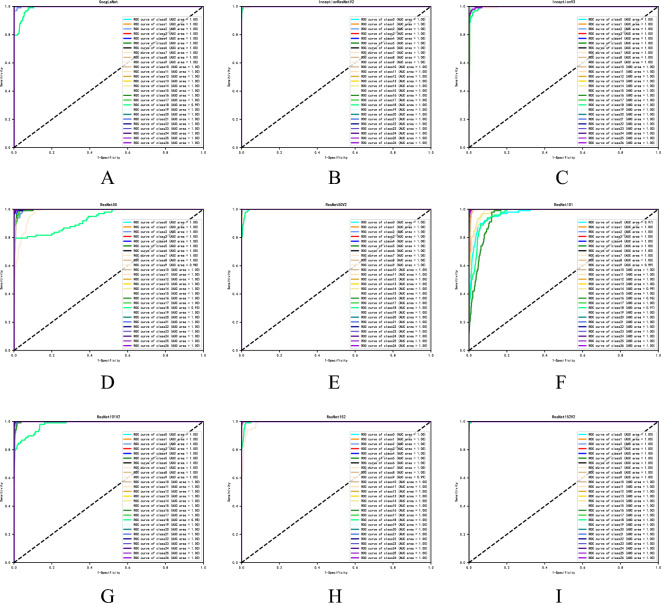
Results of ROC curves for 9 algorithms. Note: As shown in this figure, the nine algorithmic models are GoogleNet, InceptionResNetV2, InceptionV3, ResNet50, ResNet50V2, ResNet101, ResNet101V2, ResNet152 and ResNet152V2, respectively; The horizontal coordinates represent the predicted category, the vertical coordinates represent the real category, and the numbers 0–26 indicate the implant classification information as in Table 1.

The closer the ROC curve is to the upper left corner of the graph, the higher the sensitivity, the lower the 1-specificity, i.e., the higher the sensitivity, the lower the false positive rate, and the larger the AUC area, the better the performance of the diagnostic method. Therefore, according to results of the ROC curves for the 9 algorithms, ResNet152V2 has the best performance, and the AUC area (Fig. 3).

### Implant 27 classification prediction

**Figure 4:**
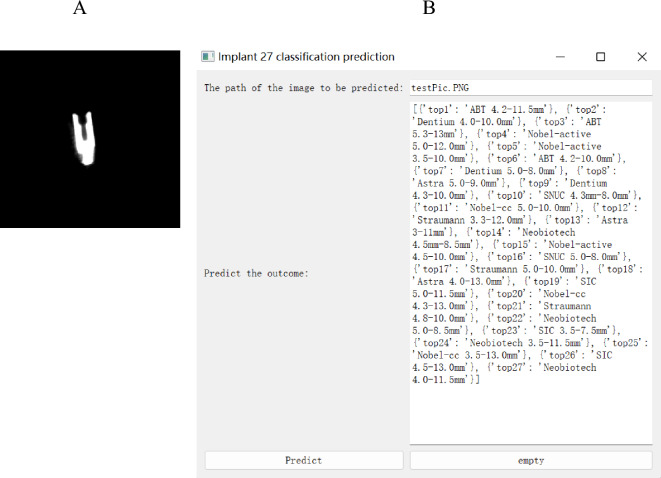
Application for implant 27 classification prediction. Note: (**A**) Implant test chart (test). (**B**) Results of the use of the implant 27 classification application developed by the Institute.

Using the optimal ResNet152V2 in conjunction with PyQt5, this study has created an application for implant 27 classification prediction, which has been tested in Affliated Stomatological Hospital of Nanchang University. As an example, the implant test image (testPic.PNG) in Fig. 4A can be entered into the program to predict the corresponding implant 27 classification probability results. The graph of the results(Fig. 4B) is obtained after inputting implant test image(Fig. 4A) into the application we developed, the probability of the corresponding prediction category decreases from front to back and the implant is most likely ABT 4.2–11.5 mm implant.The application recognizes the procedure in just a few seconds and is quite effective.

## Discussion

With the widespread use of implant restorative modalities, their maintenance and repair work is increasing, and the lack of implant-related data for various reasons makes it very difficult for dentists to identify implants through intraoral and imaging examinations. This work aims to discover a system that can accurately and rapidly identify implants using limited data. Lee et al.^22^ stated that the accuracy of using the appropriate deep learning model for implant classification from medical imaging data exceeded that of the participating dentists, and the feasibility of deep learning in implant identification has been initially verified.

Sukegawa et al.^23,24^ reported that convolutional neural networks were employed in earlier research for implant classification and identification, but they relied on image data from apical and panoramic and did not yet have a method for processing and using CBCT 3D images for implant identification. By manually intercepting the CBCT images of the implants in various levels and directions, the challenge of processing and transferring 3D data was solved in this study. The 3D images were successfully transformed into 2D data sets and trained for recognition. The sagittal and coronal were successfully located using this manual interception technique, and the resulting 2D images can prevent anatomical structure overlap and image distortion^25^.The effective processing and application of CBCT 3D images has improved the accuracy of the deep learning algorithm mode for identifying and classifying the brand and specifications of dental implants, which can assist dentists in their professional job more effectively. Additionally, this study used a total of 27 classifications and 9 brands of implants with different diameters and lengths, increasing the generalizability of the deep learning algorithm and application software in comparison to the experiment using only 12 implants in the study by Sukegawa et al.^24^. At the same time, the experiment obtained a two-dimensional data set of 13,500 images by processing 27 classified implant 3D images, which further enriched the database of implant image data and paved the way for future research on more classified implant recognition.

The nine algorithms used in this study are based on three currently popular architectures: Inception, ResNet, and a combination of Inception and ResNet, Which neural network architectures perform the best for current image categorization tasks. GoogLeNet (InceptionV1) and ResNet demonstrated excellent multi-category image classification and target detection performance in the ImageNet competition in 2014, and the ILSVRC in 2015, respectively. The 9 algorithms also achieved better results in this study, with accuracy rates above 80% in all cases. Compared to Jae-Hong Lee et al., who used only the InceptionV3 algorithm and Shintaro Sukegawa et al., who used only the ResNet system variant structure for implant recognition, we used the most algorithms and used a more comprehensive classification algorithm^20,24^.

The experimental results confirmed the accuracy of all nine deep learning algorithms for implant system identification and classification using CBCT images, especially the ResNet152V2 showed the highest classification accuracy, best accuracy in confusion matrix, and the best classification performance for the ROC curve. Although this algorithm has a large number of model parameters and a long training time, the time difference between the application developed based on it and other algorithms used for identification is short when used in clinical applications. This elapsed time is negligible when considered from the perspective of clinical applications. Considering that the best results were achieved using this algorithm for the identification of implants on CBCT images, it can better assist the dentist in identifying the implant system. This may be due to ResNet contains 12 layers of network levels, which is more favourable to picture feature extraction and better network expression. It also rebuilt a residual network's fundamental unit, making it easier to train and more effective in generalization. In addition, the findings of Figs. 3, 4 indicate that the deep learning system accurately identifies all types of implants on CBCT images. In particular, ABT 4.2–11.5 mm, Astra 5.0–9.0 mm, Nobel-CC 5.0–11.0 mm, SIC 4.5–13.0 mm, and SNUC 4.3–8.0 mm implants were best identified and basically not misclassified. The Straumann 5.0–10.0 mm implant had the worst recognition result. This result may be the consequence of a tiny change in diameter, length, and general morphology between this implant and other implants of the same brand, making it more difficult to identify than other implants.

In addition, our ultimate goal is to use deep learning algorithms for implant identification in clinical care. Through the comprehensive consideration of various evaluation indicators, the recognition effect of ResNet152V2 is optimal, therefore we use this algorithm to further develop an application for implant 27 classification that can be operated by doctors and can be augmented for reinforcement learning, and has been initially tested in Affliated Stomatological Hospital of Nanchang University. In the test process, we can directly capture and input images according to the method in this study for implants that have not completed the implantation process in other hospitals, or do not have a prosthetic components. However, for implants with a prosthesis or healing screw, only the root portion of the implant should be captured in CBCT to complete the acquisition of input images. However, the accuracy of implant identification is decreased in this way.

This study also has some limitations. First of all, the data collected in this study were from implants placed in vitro model, and the influence of background structures such as bone and the Suprastructure of the implant on the experimental results was not considered. Although we have applied the developed application to the clinic for preliminary trials, the accuracy has decreased, and there is still a lack of specific data to support this. In addition, our processing of CBCT images is still limited to two-dimensional interception and has not completed three-dimensional transformation. Therefore, in future studies, we should also address the 3D transformation of CBCT images and and collect CBCT images of patients with implants with superstructure as a dataset to complete the optimization of the application by training the dataset. Secondly, in the experimental design, we selected implants with different diameters and lengths, mainly to verify whether different types of implants of the same brand can be effectively identified. However, it is not comprehensive enough to include all types of implants. Future research will further solve this problem.Thirdly, due to the limitation of time and conditions, the CBCT images used for identification in this study were all taken by the same equipment, while the spatial resolution and magnification of different CBCT equipment differed.Therefore, in future studies, we should also conduct multicenter studies to collect more image data using different models of CBCT machines to establish a more complete and comprehensive database for better generalization of experimental results. Finally, in this study, the algorithm used in the currently developed implant 27 classification prediction is the optimal one, while the correct rate of classification may decrease with the increase of implant dataset in the future. We should use more algorithms to train the dataset and further optimize the application to make the operation easier and the classification recognition more accurate for the physician to obtain the missing implant information to assist the dentist’s clinical work.

## Conclusion

In this study, the artificial intelligence recognition system built on ResNet152V2 and the preliminary developed application software can efficiently and accurately identify the brand and specification of 27 classified implants by the processed CBCT 3D images in vitro with high stability, low recognition cost and wide applicability. The program can assist the dentist in implant identification, saving a great deal of time and increasing accuracy, which can serve as a reference for the replacement and repair of implants in the future. At the same time, the dataset of this study solves the problem of processing and transferring 3D images, further enriching the database of different types of implant images, and paving the way for future promotion of the application and for studies related to the identification of more kinds of implants.

## Data Availability

The datasets generated and analysed during the current study are available in the [Github] repository, [You can click this weblink to datasets: https://github.com/AIdental/27-/blob/main/first%20gitHub.txt].
